# Evaluation of self-administered antigen testing in a college setting

**DOI:** 10.1186/s12985-022-01927-7

**Published:** 2022-12-01

**Authors:** Sarah C. Tinker, Jessica L. Prince-Guerra, Kelly Vermandere, Jenna Gettings, Cherie Drenzik, Gary Voccio, Tonia Parrott, Jan Drobeniuc, Tonya Hayden, Stephen Briggs, Debbie Heida, Natalie Thornburg, Lisa C. Barrios, John C. Neatherlin, Sabrina Madni, Catherine N. Rasberry, Kenneth D. Swanson, Azaibi Tamin, Jennifer L. Harcourt, Sandra Lester, Lydia Atherton, Margaret A. Honein

**Affiliations:** 1 COVID-19 Response Team, Centers for Disease Control and Prevention (CDC), 1600 Clifton Rd NE, Atlanta, GA 30333 USA; 2 Laboratory Leadership Service, CDC, Atlanta, GA USA; 3 Georgia Department of Public Health, Atlanta, GA USA; 4 Epidemic Intelligence Service, CDC, Atlanta, GA USA; 5 Georgia Public Health Laboratory, Atlanta, GA USA; 6 Berry College, Rome, GA USA

**Keywords:** COVID-19, SARS-CoV-2, Institutions of higher education (IHE), Adolescent, Young adults, Quidel QuickVue at-Home OTC COVID-19 Test, Self-administered antigen test, Viral culture

## Abstract

**Background:**

The objective of our investigation was to better understand barriers to implementation of self-administered antigen screening testing for SARS-CoV-2 at institutions of higher education (IHE).

**Methods:**

Using the Quidel QuickVue At-Home COVID-19 Test, 1347 IHE students and staff were asked to test twice weekly for seven weeks. We assessed seroconversion using baseline and endline serum specimens. Online surveys assessed acceptability.

**Results:**

Participants reported 9971 self-administered antigen test results. Among participants who were not antibody positive at baseline, the median number of tests reported was eight. Among 324 participants seronegative at baseline, with endline antibody results and ≥ 1 self-administered antigen test results, there were five COVID-19 infections; only one was detected by self-administered antigen test (sensitivity = 20%). Acceptability of self-administered antigen tests was high.

**Conclusions:**

Twice-weekly serial self-administered antigen testing in a low prevalence period had low utility in this investigation. Issues of testing fatigue will be important to address in future testing strategies.

**Supplementary Information:**

The online version contains supplementary material available at 10.1186/s12985-022-01927-7.

## Background

The COVID-19 pandemic caused disruptions to institutions of higher education (IHEs). People living and working in IHEs are at increased risk of infection with SARS-CoV-2, the virus that causes COVID-19 [1], and IHE-related outbreaks have been associated with increasing spread in the surrounding communities [2]. However, cancelling in-person instruction and extracurricular activities can adversely affect students’ academic progress and mental health and can have a financial impact for students, staff, and institutions [3]. COVID-19 vaccination remains the most effective prevention strategy [4]. However, varying vaccination rates at IHEs, combined with the emergence of highly transmissible variants of SARS-CoV-2, underscore the need for continued implementation of layered prevention strategies at IHEs, including testing [1].

Data suggest that serial screening testing of asymptomatic persons may be better at preventing transmission than symptom-based or entry testing [5, 6]. One option for serial screening testing is self-administered rapid antigen tests. There is limited information on performance of these tests, their utility in controlling the spread of COVID-19 at IHEs, and their acceptability to staff and students.

We investigated the use of the Quidel QuickVue At-Home COVID-19 Test (https://quickvueathome.com/) in an IHE setting during a period of moderate to low community transmission. Our goals were to assess implementation of twice-weekly serial self-administered antigen testing among students and staff and estimate the performance of self-administered rapid antigen testing compared to real-time reverse transcription polymerase chain reaction (rRT-PCR) and seroconversion; and assess acceptability of self-administered antigen testing.

## Methods

This investigation was a collaboration of the participating college, the Georgia Department of Public Health (GDPH), and the U.S. Centers for Disease Control and Prevention (CDC). The protocol for this investigation was reviewed by their Institutional Review Boards and determined to be non-research and was conducted consistent with applicable federal law and CDC policy as defined in 45 CFR46.102(I)(2).

### Setting and Participants

The investigation was conducted between February 22nd–April 20th, 2021 at a primarily residential college in Georgia, USA. The level of COVID-19 transmission in the surrounding county was moderate at the beginning of the investigation and low at the end [7]. At the beginning of the investigation period when vaccine eligibility was limited 14.6% of the total county population had received at least one dose of COVID-19 vaccine and 9.1% were considered fully vaccinated [7]. On March 25th vaccine eligibility was expanded to everyone ≥ 16 years. By the end of the investigation, 28.7% of the county population had received at least one dose of the COVID-19 vaccine, and 22.2% were considered fully vaccinated [7]. The vaccination rate at the college at the beginning of the investigation was 19.8% of staff and 5.4% of students. Mass vaccination events were held on campus the weeks of March 22nd and April 19th; by the end of the investigation, vaccination rates among staff and students were 75.6% and 62.2%, respectively.

Most students (90%) attending the college live in residence halls on the controlled-access campus. The school instituted several COVID-19 prevention strategies, including mask mandates, physical distancing in classrooms, enhanced facility cleaning, limiting campus access, and encouraging students to form small, mutually exclusive social “bubbles” of ≤ 5 students. Students attending the college (N = 1982), staff/faculty (N = 643), and affiliates (e.g., spouses of staff) associated with the college were eligible for the investigation. Any person who was in quarantine due to SARS-CoV-2 exposure or who had COVID-19 symptoms was not eligible for testing. In the two weeks before the investigation, seven COVID-19 cases were reported on campus (prevalence: 133.3/100,000).

### Twice-weekly self-administered antigen screening testing

The Quidel QuickVue At-Home COVID-19 Test (hereafter self-administered antigen test) is a lateral flow assay that relies on qualitative detection of the SARS-CoV-2 nucleocapsid protein. Results are read visually on a test strip after ten minutes. At the time this investigation began this test was under FDA review for Emergency Use Authorization (EUA) for self-administration and received an EUA in March 2021 [8].

**Fig. 1:**
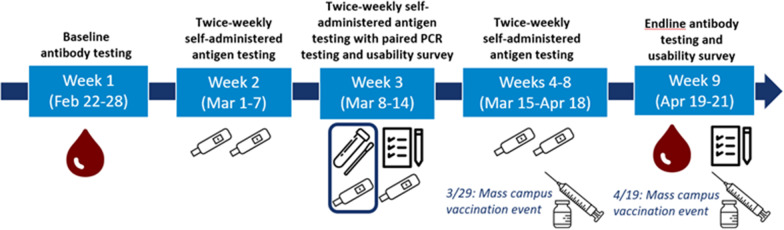
Timeline of serial self-administered COVID-19 antigen testing investigation

Participants were provided with self-administered antigen tests and manufacturer instructions and instructed to test twice weekly for seven consecutive weeks. During week three, nasal swabs for paired rRT-PCR testing were collected on the same day as antigen testing (Fig. 1).

Participants were asked to submit their self-administered antigen test results and a photo of their test strip through the college’s symptom screening online reporting system. Participants who tested positive by self-administered antigen test were counseled to obtain same-day confirmatory testing by RT-PCR and to self-isolate.

### SARS-CoV-2 IgG antibody testing

To evaluate the number of SARS-CoV-2 infections the serial twice-weekly self-administered antigen testing missed, we examined SARS-CoV-2 specific IgG seroconversion, with paired baseline (week 1) and endline (week nine) serology testing (Fig. 1). The college provided participant COVID-19 vaccination records and positive SARS-CoV-2 test results within 90 days prior to and during the investigation.

Nurses collected blood specimens from consenting participants into K2-EDTA tubes. Plasma was separated from whole blood by centrifugation within 24 h of collection. Plasma specimens were aliquoted into Nalgene cryogenic vials, heat-treated at 56 °C (132.8°F) for 10 min, and frozen at − 80 °C. One plasma aliquot was tested using the qualitative VITROS anti-SARS-CoV-2 total antibody in vitro diagnostic test on the automated VITROS 3600 Immunodiagnostic System (Ortho Clinical Diagnostics), which measures total SARS-CoV-2 antibodies against the SARS-CoV-2 spike protein*.* An automatically calculated ratio of test sample signal to cutoff value (S/C) < 1.0 was interpreted as nonreactive, and S/C ≥ 1.0 was interpreted as reactive for anti-SARS-CoV-2 total antibody.

To delineate infection versus vaccine induced antibody responses among vaccinated participants, plasma specimens were analyzed with V-plex SARS-CoV-2 panel 2 IgG kit (Meso Scale Diagnostics [MSD]) as directed by the manufacturer. This multiplex assay detects antibodies against SARS-CoV-2 nucleocapsid (N), spike (S), and the spike receptor binding domain (RBD). Specimens that were positive for nucleocapsid antibodies, in addition to S and/or RBD, were classified as having infection induced antibody response; endline specimens positive only for S and/or RBD were classified as having vaccination induced antibody response only (i.e., no evidence of infection).

### Paired rRT-PCR testing

During the third week of the investigation, participants were asked to submit a nasal swab specimen for rRT-PCR testing and to take a self-administered antigen test on the same day. Participants were directed to self-collect a bi-lateral anterior nasal swab for rRT-PCR testing. These swabs were stored in tubes with viral transport media in coolers with cold packs and transported daily to the Georgia Public Health Lab and stored at 4 °C until testing. Within 48 h of collection, specimen nucleic acid was isolated using the Perkin Elmer Chemagic Viral DNA/RNA assay on the Perkin Elmer Chemagic 360 instrument (Perkin-Elmer) and analyzed using the CDC Influenza SARS-2 (Flu SC2) Multiplex assay according to the manufacturer’s instructions for use [9]. Residual frozen specimens positive for SARS-CoV-2 by either test underwent viral culture. Specimens were cultured by limiting dilution in Vero E6/TMPRSS2 cells and were observed daily for cytopathic effects in 96-well plates, described previously [10]. Supernatant from cells that exhibited cytopathic effects was harvested and tested by rRT-PCR using the Flu SC2 Multiplex assay to confirm the presence of SARS CoV-2. A specimen was culture-positive if the first viral passage had a cycle threshold (Ct) value at least two Ct values lower than the clinical specimen.

### Acceptability and use surveys

Participants were administered surveys during weeks two and eight of the investigation to assess characteristics and acceptability of twice-weekly self-administered antigen testing via an online survey platform (Qualtrics). Participation in the 2nd survey was low and results are not reported. Survey questions are included in Additional file 1.

### Statistical analysis

Analyses were conducted using SAS (version 9.4; SAS Institute) and verified by an independent analyst. Frequencies of descriptive variables were calculated among groups of participants. Sensitivity, specificity, positive predictive value, and negative predictive value were calculated for paired self-administered antigen testing compared to rRT-PCR testing and seroconversion. Percentages were calculated for testing circumstances and acceptability of testing methods from the surveys.

## Results

### Serial self-administered antigen testing

**Fig. 2:**
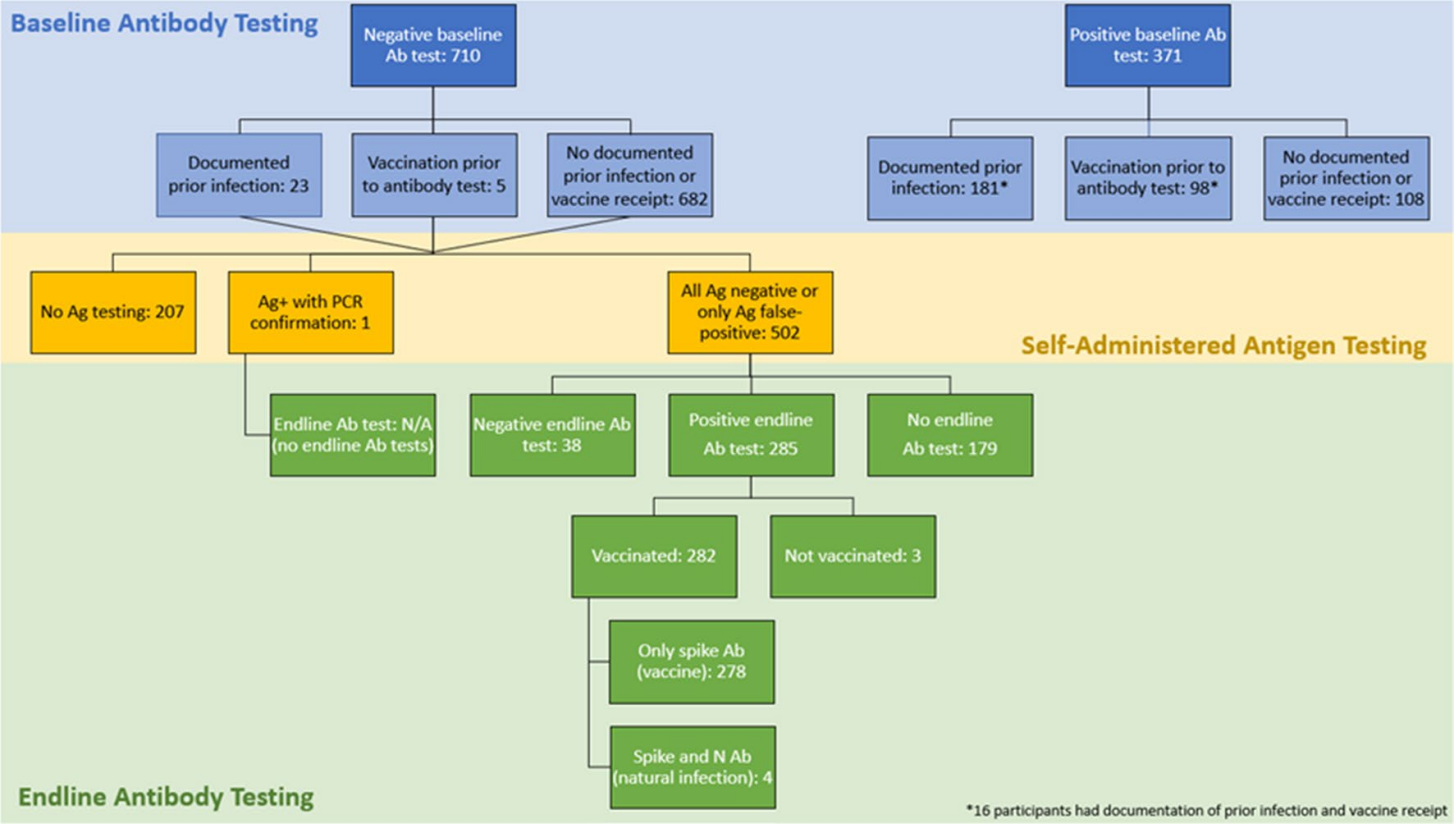
Baseline serology results for SARS-CoV2 antibodies and self-administered antigen test and endline serology results among participants who were baseline negative. Ab, antibody test; Ag, antigen test; PCR, reverse transcription polymerase chain reaction

Baseline serology results were available for 1081 participants, of which 34.3% (N = 371) tested positive for antibodies to the SARS-CoV-2 spike protein (Fig. 2). Previous infections since August 2020 were reported for 204 participants with baseline serology available; 181 (88.7%) tested positive and 23 (11.3%) tested negative. In addition, 103 participants received at least one vaccine dose prior to baseline serum specimen collection; 98 had detectable anti-S antibodies (95.2%; including 16 who also had a history of past infection), and the remaining five vaccinated participants who tested antibody negative received their first dose less than a week prior. There were 108 participants who were baseline antibody positive who did not have a record of previous infection or vaccination.

**Fig. 3:**
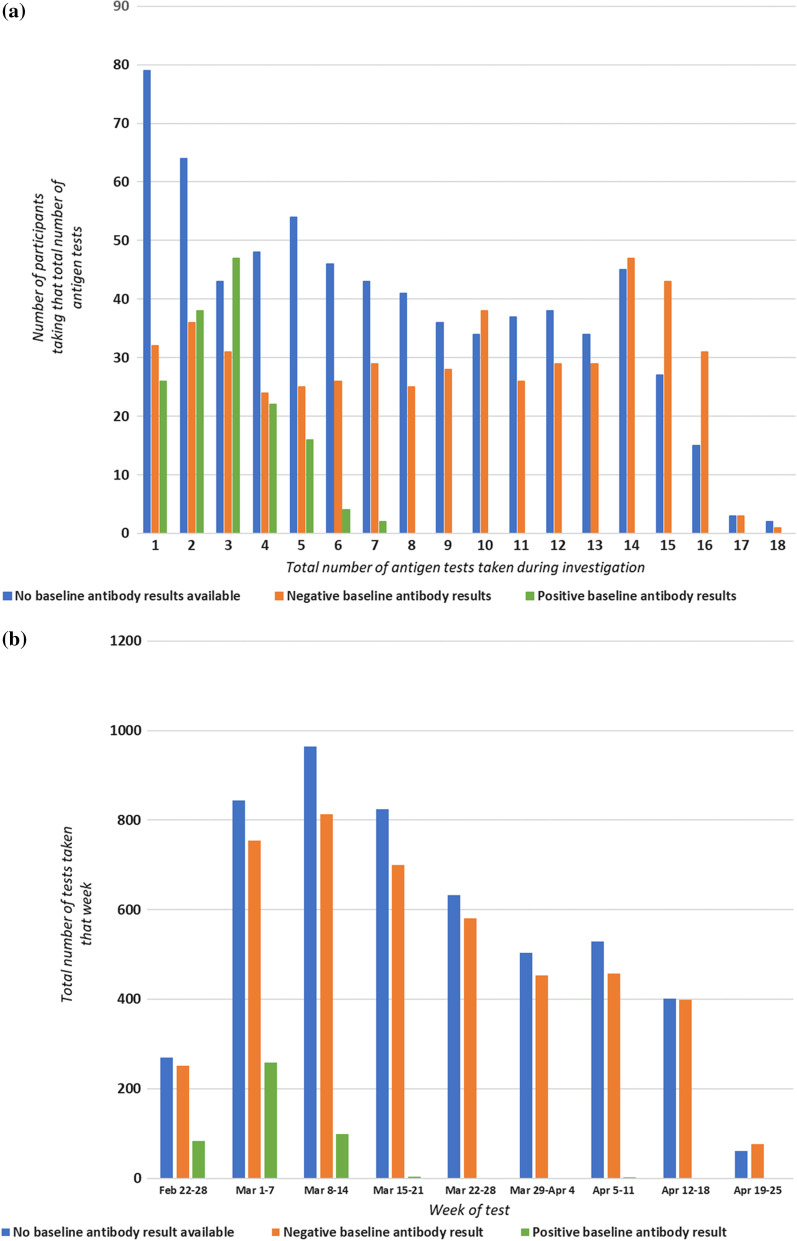
**a** Distribution of total number of self-administered COVID-19 antigen tests reported per participant during the investigation period stratified by baseline antibody status. **b** Total number of self-administered COVID-19 antigen tests by week of the investigation stratified by baseline antibody status

Results from ≥ 1 self-administered antigen test were reported by 1347 participants (Table 1); 983 students (73.0%), 363 staff (27.0%), and one affiliate (0.1%). A total of 9971 self-administered antigen test results were reported. Although participants were asked to test twice weekly for seven weeks, only 217 reported results from ≥ 14 tests (16.1%). The distribution of the total number of tests varied by baseline antibody status (Fig. 3a). Among 155 participants who were baseline antibody positive, most (~ 86%) reported taking ≤ 4 tests, particularly after results of baseline antibody testing were returned (March 8–14; Fig. 3b). Among participants who were either baseline antibody negative (n = 503) or who did not participate in baseline testing (n = 689), the median number of tests taken was eight. Participation attrition was observed among these groups as well, with the number of self-administered antigen test results reported peaking during the first two weeks of March 2021 and then steadily decreasing (Fig. 3b).

**Table 1:** Characteristics of asymptomatic college students and staff participating in investigation of self-administered antigen testing, Georgia, February–April 2021

	Serial self-administered antigen testing participants N (%)	Participants with negative baseline antibody results who participated in self-administered antigen testing, and endline antibody testing N (%)	Paired testing participants^a^ N (%)
Total	1347	324	665
Participant type			
Student	983 (73.0)	189 (58.3)	449 (67.5)
Staff	363 (27.0)	135 (41.7)	216 (32.5)
Affiliate^b^	1 (0.0)	0	0 (0.0)
Age			
< 18	6 (0.5)	0	1 (0.2)
18–22	963 (71.5)	189 (58.3)	443 (66.6)
23–34	75 (5.6)	25 (7.7)	45 (6.8)
35–49	135 (10.0)	53 (16.4)	75 (11.3)
50–64	142 (10.5)	56 (17.3)	90 (13.5)
≥ 65	26 (1.9)	1 (0.3)	11 (1.7)
Sex			
Male	392 (29.1)	97 (29.9)	190 (28.6)
Female	955 (70.9)	227 (70.1)	475 (71.4)
Race/ethnicity			
Non-Hispanic White	1072 (79.6)	262 (80.9)	533 (80.5)
Non-Hispanic Black	72 (5.4)	18 (5.6)	32 (4.8)
Hispanic	105 (7.8)	22 (6.8)	48 (7.3)
Asian	33 (2.5)	9 (2.8)	20 (3.0)
Non-Hispanic Multiracial	47 (3.5)	8 (2.5)	26 (3.9)
Other race/ethnicity	9 (0.7)	1 (0.3)	1 (0.2)
Unknown	9 (0.7)	4 (1.2)	2 (0.3)
Type of housing (students only)			
On campus housing	873 (88.8)	177 (93.7)	25 (5.6)
Off campus housing	110 (11.2)	12 (6.4)	424 (94.4)

^a^Paired testing comparing results of self-administered antigen test to results of real-time reverse transcription polymerase chain reaction from specimens taken on the same day

^b^Individuals affiliated with the college who are neither students nor staff; e.g., spouses of staff

A total of 11 participants reported positive self-administered antigen test results, all of whom received RT-PCR testing within 24 h; three infections were confirmed through this testing (27%). Baseline antibody results were available for 4/11; one was seropositive and three were seronegative.

There were 324 individuals seronegative at baseline who participated in self-administered antigen testing and endline antibody testing (Table 1, Fig. 1). Among these individuals, there were three positive self-administered antigen tests, only one of which was confirmed by RT-PCR (positive predictive value [PPV] = 33%). Of the two not confirmed by RT-PCR, 1 person tested antibody negative at endline and the other had antibodies consistent with vaccination. Three additional individuals who never reported a positive self-administered antigen test or had any record with the school of COVID-19 vaccination were antibody positive at endline; these participants reported one, two, and seven total self-administered antigen test results. However, two of these individuals tested positive only for spike protein antibodies, and not nucleocapsid antibodies, suggesting they may have been vaccinated and this information was not provided to the school. The third individual had negative results for the spike and nucleocapsid protein antibodies using the multiplex assay, but they were positive by the qualitative VITROS anti-SARS-CoV-2 total antibody in vitro diagnostic test; the reason behind these inconsistent results is not clear. An additional four individuals who never reported a positive self-administered antigen test and who received at least one dose of COVID-19 vaccination were positive for spike and nucleocapsid protein antibodies, consistent with a SARS CoV-2 infection. Based on the five COVID-19 infections with complete and consistent data, the sensitivity of the implemented serial self-administered antigen testing was 20% (1/5). When we considered the subgroup of 186 participants who reported results from ≥ 10 self-administered antigen tests, there were no positive antigen tests and there were three missed infections based on seroconversion.

### Paired testing

There were paired self-administered antigen and rRT-PCR specimens for 665 participants, 449 (67.5%) students and 216 (32.5%) faculty or staff (Table 1). Four rRT-PCR specimens were positive for SARS-CoV-2 (prevalence = 0.60%) (Table 2). Paired self-administered antigen tests were positive for two of these individuals (sensitivity = 50%). No specimens were positive by self-administered antigen test and negative by rRT-PCR (specificity = 100%). The PPV of a single self-administered antigen test compared to rRT-PCR was 100% and the negative predictive value was 99.7%. No specimens were positive for Influenza A or B.

**Table 2:** Results of paired testing for SARS-CoV-2 infection among asymptomatic students and staff at a Georgia college, March 2021

	rRT-PCR result		
	Positive	Negative	Total
Paired self-administered antigen test result			
Positive	2	0	2
Negative	2	661	663
Total	4	661	665
Sensitivity: 50% (95% CI^a^: 6.8, 93.2)		Specificity: 100% (95% CI^a^: 99.4, 100)	
PPV: 100%		NPV: 99.7% (95% CI^b^: 99.2, 99.9)	

CI estimated using: MedCalc Software Ltd. Diagnostic test evaluation calculator. https://www.medcalc.org/calc/diagnostic_test.php (Version 20.009; accessed July 28, 2021)

rRT-PCR, real-time reverse transcription polymerase chain reaction; CI, confidence interval; PPV, positive predictive value; NPV, negative predictive value

^a^Exact Clopper-Pearson Confidence Intervals

^b^Standard logit confidence intervals[16]

The Ct values for the two concordant specimens were 18.5 and 19.4, while for discordant specimens they were 33.5 and 35.6. Culturable virus was detected in 1/4 (25%) rRT-PCR positive specimens. This specimen had a Ct value of 18.5 and was also positive by the self-administered antigen test (sensitivity = 100% among culture positive specimens). Both participants with concordant specimens developed symptoms; the two participants with discordant specimens were asymptomatic.

### Acceptability and use of twice-weekly self-administered antigen testing survey results

Among participants who submitted antigen test results, 753 (641 students, 112 staff) completed the survey (Table 3). Most students reported taking the self-administered tests alone in their dorm rooms, and few reported problems taking the test (0.5%). Almost half of students reported RT-PCR testing with a nasal swab specimens as their preferred COVID-19 test type (46.3%), but approximately 1/3 of students reported preferring self-administered antigen testing (35.7%). Results were similar for staff, who were also likely to take the test alone and reported few problems taking the test (Table 3).

**Table 3:** Results of survey assessing characteristics and acceptability of serial self-administered antigen testing at a Georgia college, March 2021

	Students	Staff
	N^1^ (%)	N^a^ (%)
Total	641	112
Location of testing		
Dorm room (student only)	417 (65.1)	N/A
Dorm room common area (student only)	0 (0.0)	N/A
Dorm bathroom (student only)	0 (0.0)	N/A
Bedroom	31 (4.8)	8 (7.1)
Bathroom	5 (0.8)	15 (13.4)
Kitchen	10 (1.6)	25 (22.3)
Living room	6 (0.9)	11 (9.8)
Work office (staff only)	N/A	17 (15.2)
Other area	10 (1.6)	19 (17.0)
Not specified	162 (25.3)	17 (15.2)
Test taken alone or with others		
Alone	404 (63.0)	98 (87.5)
With other people	60 (9.4)	8 (7.1)
With only one other person in the room	39 (65.0)	6 (75.0)
With one other person taking the test	30 (50.0)	5 (62.5)
Missing	177 (27.6)	6 (5.4)
Problems taking the test		
None	509 (79.4)	104 (92.9)
Any^b^	3 (0.5)	3 (2.7)
Missing	129 (20.1)	5 (4.5)
Preferred type of COVID-19 test		
PCR—saliva	49 (7.6)	3 (2.7)
PCR—nasal swab	297 (46.3)	51 (45.5)
Antigen test done by healthcare professional	31 (4.8)	5 (4.5)
Antigen test done by themselves at home	229 (35.7)	52 (46.4)
Missing	35 (5.5)	1 (0.9)
Most difficult part of performing the home antigen test		
Nasal swab collection	142 (22.2)	13 (11.6)
Performing the test using the test card	85 (13.3)	9 (8.0)
Reading the results of the test card	49 (7.6)	7 (6.3)
Reporting the results	157 (24.5)	33 (29.5)
Other	120 (18.7)	38 (33.9)
Missing	88 (13.7)	12 (10.7)
I like using home antigen testing every week to reduce the spread of COVID-19		
Completely agree or agree	403 (67.9)	72 (64.9)
Neither agree nor disagree	132 (22.2)	29 (26.1)
Disagree or completely disagree	59 (9.9)	10 (9.0)
The amount of time I spent completing the home antigen test was manageable		
Completely agree or agree	512 (86.9)	100 (90.1)
Neither agree nor disagree	70 (11.9)	9 (8.1)
Disagree or completely disagree	7 (1.2)	2 (1.8)
Home antigen testing every week will have an impact on reducing the spread of COVID-19 at [my] College		
Completely agree or agree	396 (66.4)	73 (65.8)
Neither agree nor disagree	144 (24.2)	27 (24.3)
Disagree or completely disagree	56 (9.4)	11 (9.9)
Home antigen testing every week will have an impact on reducing the spread of COVID-19 in the surrounding community		
Completely agree or agree	371 (62.3)	67 (60.4)
Neither agree nor disagree	163 (27.4)	32 (28.8)
Disagree or completely disagree	62 (10.4)	12 (10.8)
Home antigen testing every week will make me less likely to catch COVID-19 from someone else		
Completely agree or agree	286 (48.4)	28 (25.2)
Neither agree nor disagree	174 (29.4)	40 (36.0)
Disagree or completely disagree	131 (22.2)	43 (38.7)
Home antigen testing every week will make me less likely to spread COVID-19 to someone else		
Completely agree or agree	445 (75.4)	75 (67.6)
Neither agree nor disagree	101 (17.1)	29 (26.1)
Disagree or completely disagree	44 (7.5)	7 (6.3)
Home antigen testing every week makes me feel more comfortable attending classes or work on campus in person		
Completely agree or agree	347 (58.7)	60 (54.1)
Neither agree nor disagree	170 (28.8)	37 (33.3)
Disagree or completely disagree	74 (12.5)	14 (12.6)

^a^Limited to participants who responded to the survey and for whom at least one self-administered antigen test result was reported; sums of response counts may not equal totals due to missing responses

^b^“Other responses” included remembering to take the test, uploading a picture of the test result, or a lack of test tube holder

The acceptability of the self-administered test was high among students and staff (Table 3). More than 2/3 reported agreeing or completely agreeing with the statement “I like using home antigen testing every week to reduce the spread of COVID-19”. More than 85% of respondents agreed or completely agreed that the amount of time spent completing self-administered antigen testing was manageable. The percentage who agreed or completely agreed that serial self-administered testing would have an impact on reducing the spread of COVID-19 at their college was 66.4% for students and 65.8% for staff. Similar results were observed for the impact on reducing spread of COVID-19 in the community. Staff were less likely than students to report that self-administered antigen testing would make them less likely to catch or spread COVID-19, but more than half of students and staff agreed or completely agreed that weekly self-administered antigen testing makes them feel more comfortable attending classes or working on campus in person.

## Discussion

Results from this investigation showed low sensitivity of serial self-administered antigen testing for detecting asymptomatic infections and an indication of a low positive predictive value (8/11; 73%); participation was also an issue given only 16% of participants submitted all 14 test results and 20% submitted ≤ 2 tests. Estimated sensitivity and specificity from paired rRT-PCR testing was 50% and 100%, respectively, although the number of rRT-PCR positive specimens was small (n = 4), leading to imprecise estimates. Survey results indicate high acceptability of self-administered antigen testing. Falling levels of community transmission and widespread availability of vaccines during the investigation likely impacted participation, although attrition was observed among participants with and without a record of COVID-19 vaccination (Additional file 2: Figure S1).

Although vaccination is an effective measure for COVID-19 prevention, a high level of vaccination and high effectiveness is required to reduce transmission. A modelling study showed that, prior to the emergence of more transmissible variants, if vaccination coverage is < 95 percent, daily mass testing of ~ 1/3 of the population is needed to safely accommodate full capacity in-person IHE attendance [11]. CDC provides risk-based guidance for testing strategies in IHE settings [4], including serial screening testing strategies. More data are needed to identify the best strategies for testing under different circumstances, including lower vaccine effectiveness.

While many RT-PCR assays are highly sensitive for COVID-19 testing, results can take days to be returned and administering tests can be resource intensive. Many antigen tests provide rapid results and can require less resources, but they are typically less sensitive. Evidence suggests that controlling COVID-19 outbreaks depends more on test frequency and reporting speed than test sensitivity [12].

Testing error is likely to be greater with self-administered antigen testing. A BinaxNOW antigen test evaluation showed lower sensitivity when the test was self-administered compared to administration by trained health care providers, but the sample size was small [13].

We observed several (8/11) false-positive antigen tests, but no clear reason was identified. User error is an unlikely explanation, as photos of the tests strips confirmed reported positive results. Some participants reporting false-positive test results demonstrated self-testing to a school official, who confirmed both positive results and proper methodology. The school official also tested themselves to confirm there was not an error with the test kits and they had a negative test result.

Serial antigen testing in this investigation did not identify all infections based on seroconversion. An assessment of the Quidel SARS Sofia antigen test administered every three days had > 98% sensitivity for identifying infected individuals, while daily screening had ~ 90% sensitivity for identifying individuals while they were viral culture positive [14]. More frequent testing or higher levels of compliance with twice weekly testing may have been needed to detect infections in our investigation. However, given that few participants completed all requested tests, compliance with additional requested testing is uncertain. Testing was not required by the college during the investigation period.

It is important that individuals receive proper guidance when using self-administered antigen tests. People positive by antigen test should be tested by RT-PCR as soon as possible and should begin isolating immediately. People who test negative by antigen test should understand the lower sensitivity of the test and that their test result does not rule out COVID-19 infection that can be spread to other people [15].

Our investigation had several limitations. The low COVID-19 prevalence during the study period led to small numbers of positives and imprecise estimates. Increasing vaccination availability during the investigation likely led to a lower prevalence of infection on campus and decreased interest in testing. Only a subset of participants who participated in serial self-administered antigen testing provided both baseline and endline serology specimens s (~ 30%).

## Conclusions

Our data suggest that twice weekly screening testing with the Quidel QuickVue At-Home COVID-19 Test may have low utility in low prevalence settings among asymptomatic individuals. While more frequent testing could improve the sensitivity to detect asymptomatic infections, motivating people to conduct serial testing could be challenging. Self-administered antigen tests may be of greater utility when used among symptomatic or individuals at higher risk, or in settings of substantial or high transmission.

## Supplementary Information

**Additional file 1: Supplementary Methods:** Survey questions to assess characteristics and acceptability of twice-weekly self-administered antigen testing.

**Additional file 2: Figure S1.** Total number of self-administered COVID-19 antigen tests by week of the investigation stratified by vaccination status.

## Data Availability

Due to the nature of this research, participants of this study did not agree for their data to be shared publicly, so supporting data is not available.

## Abbreviations

CDC: U.S. Centers for Disease Control and Prevention
Ct: Cycle threshold
EUA: Emergency Use Authorization
GDPH: Georgia Department of Public Health
IHE: Institutions of higher education
PPV: Positive predictive value
RBD: Receptor binding domain
rRT-PCR: Real-time reverse transcription polymerase chain reaction
