# Supplementary material for: Evaluation of self-administered antigen testing in a college setting
Source: Virol J. 2022 Dec 1;19:202. doi: 10.1186/s12985-022-01927-7 (PMC9713151; doi:10.1186/s12985-022-01927-7)
Supplement: Supplementary file 1 — Additional file 1: Supplementary Methods: Survey questions to assess characteristics and acceptability of twice-weekly self-administered antigen testing. [file 12985_2022_1927_MOESM1_ESM.docx]

SUPPLEMENTARY METHODS

*Survey questions to assess characteristics and acceptability of twice-weekly self-administered antigen testing*

Questionnaire 1: Administered Week 2

1. Have you taken at least one home antigen test for COVID-19 infection in the last 7 days?
   1. YES 🡪 continue to Question 2
   2. NO 🡪 Please select the reason why you have not taken at least one home antigen test for COVID-19 infection in the last 7 days [*Select all that apply*]
      1. Tested positive for COVID-19 in the 3 months before this investigation began
         1. *Note: remove this option if these individuals are not excluded*
      2. Tested positive for antibodies to COVID-19
         1. *Note: remove this option if these individuals are not excluded*
      3. Did not have any antigen test kits
      4. Did not understand how to use the antigen tests
      5. Forgot to test
      6. Decided I did not want to test
      7. Gave my tests to someone else
      8. Other
         1. Please specify
      9. *If you do not have antigen test kits or are having trouble using the tests, please contact [phone number] or [email] for assistance.*
      10. **SKIP TO Question 6**
2. What date did you take your most recent home antigen test?
3. Where have you taken the home antigen test? [*Select all that apply*]
   1. Dorm room
   2. Dorm common area
   3. Dorm bathroom
   4. Bedroom
   5. Bathroom
   6. Kitchen
   7. Living room
   8. Other
      1. Please specify
4. Do you take the home antigen test alone or with other people? [*Select all that apply*]
   1. Alone
   2. With other people
      1. If “With other people”: How many other people are usually present when you take your test?
      2. If “With other people”: How many other people are usually taking their test at the same time you take your test?
5. Did you have any problems taking the home antigen test?
   1. NO
   2. YES 🡪 Please select the problems that you had [*Select all that apply*]
      1. Did not understand how to use the nasal swab to take a sample
      2. Did not understand how to test the nasal swab sample
      3. Did not understand how to read the results of the test
      4. Test did not work as I expected
      5. Used the test incorrectly
      6. Problems with the testing materials (for example, the swab was broken)
      7. Other
         1. Please describe
6. On average, how long did it take you to complete the home antigen test?
7. What type of SARS-CoV-2 testing do you prefer?
   1. Saliva collection for PCR testing
   2. Nasal swab collection for PCR testing
   3. Antigen testing (done by a health care professional)
   4. Antigen testing (done by myself at home)
8. Why do you prefer this type of test?
9. On a scale of 1 – 10, how challenging did you find using the home antigen test, with 1 being easy and 10 being too difficult to perform?
10. What was the most difficult part of performing the home antigen test?
    1. Nasal swab collection
    2. Performing the test using the swab in the test card
    3. Reading the results of the test card
    4. Reporting the results
    5. Other
       1. Specify

For the following statements please indicate to what extent you agree with the statement.

1. I like using home antigen testing every week to reduce the spread of COVID-19.
   1. Completely agree
   2. Agree
   3. Neither agree nor disagree
   4. Disagree
   5. Completely disagree
2. The amount of time I spent completing the home antigen test was manageable.
   1. Completely agree
   2. Agree
   3. Neither Agree nor disagree
   4. Disagree
   5. Completely agree
3. Home antigen testing every week will have an impact on reducing the spread of COVID-19 at my college.
   1. Completely agree
   2. Agree
   3. Neither agree nor disagree
   4. Disagree
   5. Completely disagree
4. Home antigen testing every week will have an impact on reducing the spread of COVID-19 in the surrounding community.
   1. Completely agree
   2. Agree
   3. Neither agree nor disagree
   4. Disagree
   5. Completely disagree
5. Home antigen testing every week will make me less likely to catch COVID-19 from someone else.
   1. Completely agree
   2. Agree
   3. Neither agree nor disagree
   4. Disagree
   5. Completely disagree
6. Home antigen testing every week will make me less likely to spread COVID-19 to someone else.
   1. Completely agree
   2. Agree
   3. Neither agree nor disagree
   4. Disagree
   5. Completely disagree
7. Home antigen testing every week makes me feel more comfortable attending classes or work on campus in person.
   1. Completely agree
   2. Agree
   3. Neither agree nor disagree
   4. Disagree
   5. Completely disagree

Questionnaire 2: Administered Week 9

1. Have you taken at least one home antigen test for COVID-19 infection in the last 9 weeks?
   1. YES 🡪 Continue to Question 2
   2. NO 🡪 Why did you not take at least one home antigen test for COVID-19 at your residence in the last 9 weeks?
2. How many home antigen tests did you take home over the last 9 weeks?
3. What date did you take your most recent home antigen test?
4. Where have you taken the home antigen test? [*Select all that apply*]
   1. Dorm room
   2. Dorm common area
   3. Dorm bathroom
   4. Bedroom
   5. Bathroom
   6. Kitchen
   7. Living room
   8. Other
      1. Please specify
5. Did you take the home antigen tests alone or with other people? [*Select all that apply*]
   1. Alone 🡪 Continue to Question 6
   2. With other people
      1. If “With other people”: How many other people are usually present when you take your test?
      2. If “With other people”: How many other people are usually taking their test at the same time you take your test?
6. Did you have any problems taking the at home antigen test?
   1. NO 🡪 Continue to Question 7
   2. YES 🡪 Please select the problems that you had [*Select all that apply*]
      1. Did not understand how to use the nasal swab to take a sample
      2. Did not understand how to test the nasal swab sample
      3. Did not understand how to read the results of the test
      4. Test did not work as I expected
      5. Used the test incorrectly
      6. Problems with the testing materials (for example, the swab was broken)
      7. Other
         1. Please describe
7. On average, how long did it take you to complete the home antigen test?
8. What type of SARS-CoV-2 testing do you prefer?
   1. Saliva collection for PCR testing
   2. Nasal swab collection for PCR testing
   3. Antigen testing (done by a health care professional)
   4. Antigen testing (done by myself at home)
9. Why do you prefer this type of test?
10. On a scale of 1 – 10, how challenging did you find using the home antigen test, with 1 being easy and 10 being too difficult to perform?
11. What was the most difficult part of performing the test?
    1. Nasal swab collection
    2. Performing the test using the swab in the test card
    3. Reading the results of the test card
    4. Reporting the results
    5. Other
       1. Specify
12. Would you be willing to continue using home antigen testing at least once every week?
    1. Yes
    2. No
       1. If no, why not?

For the following statements please indicate to what extent you agree with the statement.

1. I like using home antigen testing every week to reduce the spread of COVID-19.
   1. Completely agree
   2. Agree
   3. Neither agree nor disagree
   4. Disagree
   5. Completely disagree
2. The amount of time I spent completing the home antigen test was manageable.
   1. Completely agree
   2. Agree
   3. Neither Agree nor disagree
   4. Disagree
   5. Completely agree
3. Home antigen testing every week will have an impact on reducing the spread of COVID-19 at my c ollege.
   1. Completely agree
   2. Agree
   3. Neither agree nor disagree
   4. Disagree
   5. Completely disagree
4. Home antigen testing every week will have an impact on reducing the spread of COVID-19 in the surrounding community.
   1. Completely agree
   2. Agree
   3. Neither agree nor disagree
   4. Disagree
   5. Completely disagree
5. Home antigen testing every week will make me less likely to catch COVID-19 from someone else.
   1. Completely agree
   2. Agree
   3. Neither agree nor disagree
   4. Disagree
   5. Completely disagree
6. Home antigen testing every week will make me less likely to spread COVID-19 to someone else.
   1. Completely agree
   2. Agree
   3. Neither agree nor disagree
   4. Disagree
   5. Completely disagree
7. Home antigen testing every week makes me feel more comfortable attending classes or work on campus in person.
   1. Completely agree
   2. Agree
   3. Neither agree nor disagree
   4. Disagree
   5. Completely disagree
