# Supplementary figures and images for: Evaluation of self-administered antigen testing in a college setting
Source: Virol J. 2022 Dec 1;19:202. doi: 10.1186/s12985-022-01927-7 (PMC9713151; doi:10.1186/s12985-022-01927-7)

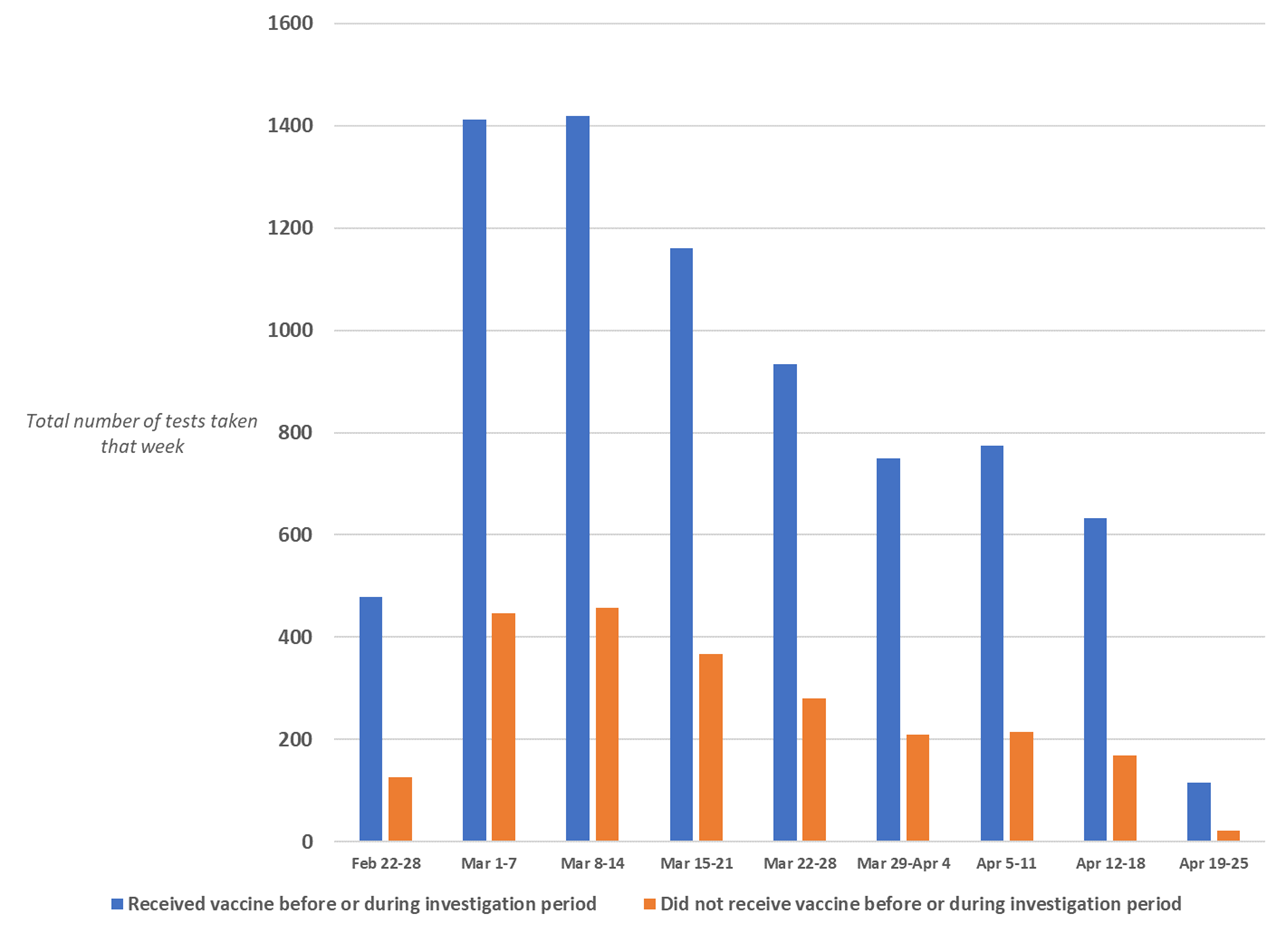

Supplement: Supplementary file 2 — Additional file 2: Figure S1. Total number of self-administered COVID-19 antigen tests by week of the investigation stratified by vaccination status. [file 12985_2022_1927_MOESM2_ESM.tif]
